# Post-Operative Abnormal Adhesions

**Published:** 1914-08

**Authors:** J. O. Kinard

**Affiliations:** 515 Empire Life Bldg., Atlanta, Ga.


### Journal-Record of Medicine

Successor to Atlanta Medical and Surgical Journal, Established 1855
and Southern Medical Record, Established 1870

**OWNED BY THE ATLANTA MEDICAL JOURNAL COMPANY**

PUBLISHED MONTHLY

*Official Organ Fulton County Medical Society, State Examining Board, Presbyterian Hospital, Atlanta, Birmingham and Atlantic Railroad Surgeons' Association, Chattahoochee Valley Medical and Surgical Association, Etc.*

EDGAR BALLENGER, M. D., Editor
BERNARD WOLFF, M. D., Supervising Editor
A. W. STIRLING, M. D., C. M., D. P. H.; J. S. HURT, B. Ph., M.D.
GEO. M. NILES, M. D., W. J. LOVE, M. D., (Ala.); Associate Editors
R. R. DALY, M. D., Assiciate Editor
E. W. ALLEN, Business Manager

**COLLABORATORS**

DR. W. F. WESTMORLAND, General Surgery
F. W. McRAE, M. D., Abdominal Surgery
H. F. HARRIS, M. D., Pathology and Bacteriology
E. B. BLOCK, M. D., Diseases of the Nervous System
MICHAEL HOKE, M. D., Orthopedic Surgery
CYRUS W. STRICKLER, M. D., Legal Medicine and Medical Legislation
E. C. DAVIS, A. B., M. D., Obstetrics
E. G. JONES, A. B., M. D., Gynecology
R. T. DORSEY, JR., B. S., M. D., Medicine
L. M. GAINES, A. B., M. D., Internal Medicine
GEO. C. MIZELL, M. D., Diseases of the Stomach and Intestines
L. B. CLARKE, M. D., Pediatrics
EDGAR PAULIN, M. D., Opsonic Medicine
THEODORE TOEPEL, M. D., Mechano Therapy
A. W. STIRLING, M. D., Etc., Diseases of the Eye, Ear, Nose and Throat
BERNARD WOLFF, M. D., Diseases of the Skin
E. G. BALLENGER, M. D., Diseases of the Genito-Urinary Organs

VOL. LXI. ATLANTA, GA., AUGUST, 1914. NO. 4

### POST-OPERATIVE ABDOMINAL ADHESIONS.

By J. O. KINARD, M. D.

I probably should say in the beginning of this paper that I do not expect to impress you or show you any new thoughts or ideas on the above subject, but I do hope to impress you, who have not had any serious trouble or experience with postoperative abdominal adhesions, and with the importance of same. You may say at the beginning or the end of any paper on this subject that the surgeon is to blame for abdominal adhesions, but I do not believe, after much experience, that you will think the surgeon is always to blame.

Post-operative abdominal adhesions are the greatest evils to abdominal surgery. Brilliant results are obtained by the removal of a diseased organ, but the immediate effect may be only temporary and the patient becomes an invalid for months, years and a lifetime as the result of adhesions.

The surgeon has been abused by his patients many a time or they may still worry him with abdominal pains, or various symptoms until he may wish he had not operated. The question of post-operative abdominal adhesions has taxed the skill and experience of the leading surgeons of the world as reports here will show what some say.

There certainly are some cases more susceptible to adhesions. Records will show that some cases have been operated on as much as 14 times for adhesions. In my own practice, I operated on one patient twice for, or as the result of, adhesions. The first operation was an appendectomy, being a female and suspecting other trouble on account of the symptoms complained of, I opened the abdomen in the median line. The appendix, which showed a chronic state of inflammation, was removed. also a cystic tumor of left ovary. One thing very noticeable about this case was, that the abdominal cavity contained a great amount of dark peritoneal fluid. The operation was not long and the intestines were handled very little, the appendix being found in the normal position. After the appendectomy the intestines were packed out of the field of operation by moist pads, wrung out of saline solution.

I might mention that there were some omental adhesions at the time of this operation. The patient was able to be out of the hospital in two weeks.

The second operation I performed on this patient was about two months later for acute intestinal obstruction. The omentum had dropped down over a loop of intestine and become attached to the posterior abdominal wall, causing a complete obstruction of the small intestine. At this operation the intestines were handled very little; adhesions were seen, but the patient's condition did not warrant further interference at this time, and we had her off the table in about 30 minutes. In about two weeks this same patient asked for another operation because she said she did not feel right and asked me what was the trouble. I told her about the adhesions and also told her that they were likely to be there after another operation, but she insisted, and I reluctantly operated. I found a complete mass of adhesions between abdominal wall, omentum and small intestines—in fact, it appeared to be a complete mass of all the small intestine and omentum. I worked for about two hours breaking up adhesions, more especially, separating the omentum from the intestines. After separating the omentum from the intestines I resected it (omentum), close up to the transverse colon, and the results have been satisfactory thus far, now over a year since the operation; the patient, an office girl, has worked steadily since she began work about one month after the operation.

I have had occasion to open the abdomen on post-operative cases of my own and others and have always found some adhesions, but the special case reported here presented conditions and complications rarely met with, I believe, and I am convinced, had I not removed the entire greater omentum close up to the transverse colon that troublesome adhesions and probably intestinal obstruction would have necessitated the opening of the abdomen ere this. The reason I say this, the omentum seemed to be the trouble maker. Reports of many prominent surgeons show more operations for adhesions than any other surgical complications. As the cause of adhesions in ordinary cases we all agree, that it is too much handling of the intestines, leaving raw surfaces not covered by peritoneum, blood clots, and sponging with dry gauze sponges, etc. After hearing what the prominent surgeons of the country say about adhesions, we might conclude that it was my technique that caused the unusual amount of adhesions, but such was not the case, because I found some adhesions at the first operation and did not encounter any inconvenience at all throughout the operation.

Dr. Murphy, of Chicago, reports a case operated on 14 times for abdominal adhesions; another doctor reports a case operated on 10 times for adhesions with even moderate relief; another physician reports a case operated on 7 times for adhesions, the last of which included intestinal resection with only partial relief.

Dr. Reichelderfer, of Washington, D. C., reports a case operated on 4 times for adhesions following a simple appendectomy. In the last three operations performed by himself for post-operative abdominal adhesions, he states that his patient was in the hospital on 10 occasions, aggregating about 250 days. On his first and second operation he tried breaking up adhesions, but after the third operation he put her in the sitting position as soon as she reacted from the anesthesia, with a tight abdominal binder and allowed her out of bed the third day. After four years the patient is doing nicely. Many a woman, I believe, has been made more miserable than benefited by operations, on account of adhesions following the opening of the abdomen; at the same time many lives have been saved by abdominal operations. I don't want it understood that I am pessimistic in making these remarks, but the time is coming, when more care and deliberation will be used before opening the abdomen than in the past. We know more and are better prepared than formerly for finding out about the condition of our patients, and we are more interested in the after-results of our operations, because we have seen our patients suffer the results of adhesions. Many say it is because their ovaries were unnecessarily removed, or their appendix was not inflamed, but not true, because they have adhesions.

I wrote to some of the leading surgeons of the country to find out what they were doing for post-operative abdominal adhesions and here I will give their replies verbatim: Dr. Chas. P. Noble says, "adhesions may be minimized by gentle handling of the intestines, by strict asepsis, avoidance of rubbing peritoneum and intestines with dry gauze, and covering of raw surfaces with peritoneum." "In septic cases adhesions are inevitable and largely conservative." Now these things the surgeon knows, if he practices that, he will be all right in most of his cases.

Dr. A. J. Ochsner said in his letter: "The only method of preventing adhesions that we are using at the present time consists in carefully covering over raw surfaces in the peritoneal cavity with peritoneum while suturing it in the line of incision. In cases in which there are extensive adhesions we have used liquid vaseline but I am not certain as to the amount of benefit we have obtained from this. At the present time we are waiting for a case in which we used a 2% citrate of soda solution and 3% sodium chloride, which has just been highly recommended." In reply to my letter to Dr. J. B. Murphy, he states: "I have found no method so far that is of any particular value in preventing abdominal adhesions except in some way to cover the abraded peritoneal areas, either by the sigmoid or some other tissue, but never by foreign material." He says, "I had a case the other day in which vaseline had been used to cover the abraded surfaces and it was the worst case of peritoneal adhesions that I had ever seen. Each mass of vaseline had one capsule and some three or four, so that it was one conglomerate mixture of vaseline, clumps of adhesions, matted intestines, etc. I think any foreign material put into the abdomen is a detriment and favors adhesions rather than prevents them."

You will notice that none of the surgeons highly recommend any foreign material in the abdomen, though some do recommend cargile membrane, the dried sterile peritoneum of the ox. Olive oil has been used, but the results have not been satisfactory, in fact, a number of things have been recommended, as in any incurable disease. They have all been tried and found wanting.

I have noticed in my abdominal work that where I open the abdomen and find abnormal peritoneal fluid, that the adhesions are greater and the patient more susceptible to all kinds of peritoneal adhesions. Previous infections may be the cause of the abnormal condition of the peritoneal fluid, though some cases I have observed with a negative history.

As to the treatment of post-operative abdominal adhesions I have nothing to offer, except the routine precautions, of carefully handling the intestines, peritoneum and other viscera, and covering all raw surfaces, and especially in cases with much congested or inflamed omentum, then resect that part that seems most liable to give after-trouble, that is, the part of the omentum that shows a tendency to adhesions. The omentum has much to do with the troublesome adhesions following abdominal surgery. I have found Eserine sulphate valuable, given in doses of gr. 1-50 to 1-100 t. i. d. In severe conditions gr. 1-50 Q. 4 Hr. I almost invariably follow any condition presenting suspicion toward adhesions with Eserine Gr. 1-100 t. i. d. Eserine in small doses does very little good. It certainly does have a tendency to create peristalsis. In cases liable to severe adhesions I give turpentine enemata t. i. d. and eserine sulphate gr. 1-100 to 1-50 t. i. d. and believe I have gotten good results. The sitting position following abdominal work may be valuable, but if the omentum is long and is not removed there will likely occur severe adhesions.

515 Empire Life Bldg., Atlanta, Ga.

REFERENCES.

Journal of Surgery, Obstetrics and Gynecology.

Kelly & Noble, Abdominal Surgery.

